# Morphological Changes One Year After Achilles Tendon Rupture Repair: A Comparison of Loading Strategies

**DOI:** 10.1155/tsm2/1778630

**Published:** 2025-09-28

**Authors:** Rikke Hoeffner, Rene B. Svensson, Syed Zahra Gillani, Frederik Hvid Linden, S. Peter Magnusson

**Affiliations:** ^1^Department of Orthopaedic Surgery, Institute of Sports Medicine Copenhagen, Copenhagen University Hospital Bispebjerg-Frederiksberg, Copenhagen, Denmark; ^2^Department of Physical and Occupational Therapy, University Hospital Bispebjerg-Frederiksberg Hospital, Copenhagen, Denmark; ^3^Department of Radiology, University Hospital Bispebjerg-Frederiksberg Hospital, Copenhagen, Denmark

## Abstract

**Purpose:** This study investigated effects of delayed initial loading in comparison to a standard regime following an Achilles tendon rupture (ATR) on tendon cross-sectional area (CSA) and fat infiltration by using 3D magnetic resonance imaging (MRI) and ultrasound (US) measurements.

**Methods:** Secondary analysis of a randomized controlled trial. Forty-eight patients with an ATR were randomized to a Standard regime with weight-bearing after 6 weeks or a Delayed regime with weight-bearing after 12 weeks postsurgery. Achilles tendon CSA, fat fraction, and vascularization were measured on both the injured and the uninjured side using MRI and US at 12 and 52 weeks.

**Results:** The injured tendon was significantly larger (> 300%) than the uninjured tendon for both the distal and proximal parts (*p* < 0.0001). The CSA of the distal part was smaller in the Delayed group at 3 months (*p*=0.038), but not at 12 months. Fat fraction in the tendon increased in both groups. The Delayed group had significantly less vascularization compared with the Standard group at 3 months, and the vascularization decreased in both groups from 3 to 12 months.

**Conclusion:** In comparison to the Standard treatment, the Delayed group had reduced CSA and vascularization for the distal part of the tendon after 3 months. After a year, these differences had become insignificant. From 3 to 12 months, the distal parts of the injured tendon showed an accumulation of fat in both groups. Not only the rupture site but also the entire tendon was affected by the inflammatory repair response.

**Trial Registration:** ClinicalTrials.gov identifier: NCT04263493.

## 1. Introduction

Over the past few decades, the incidence of acute Achilles tendon ruptures (ATRs) has increased and it is currently estimated to be 31–35 per 100,000 persons annually in the Scandinavian countries [1–3], which may be related to an aging and physically active population. Both men and women can sustain an ATR, but middle-aged men (30–50 years old) who occasionally participate in sports are particularly at increased risk of sustaining the injury [1]. The optimal rehabilitation strategy following ATR repair remains a matter of debate. However, recent literature suggests that an accelerated rehabilitation protocol has become a standard [4–6]. Despite efforts to improve outcomes following ATR, it frequently results in long-term functional limitations [7], and factors such as tendon cross-sectional area (CSA), surgical or nonsurgical treatment, muscle atrophy, heel-rise function, age, and body mass index have all been suggested to predict a patient's prognosis [8–12].

The CSA of the healed Achilles tendon will typically be much larger post ATR injury [12, 13], which may be influenced by the healing response during tissue restoration [13, 14]. There are three overlapping phases to tendon healing [15–17]. There is an initial hematoma in the wounded area in which inflammatory cells and platelets are activated that initiates an acute inflammatory process [16]. The second phase begins a few days later by formation of granulation tissue by the tenocytes and inflammatory cells. Collagen type III is produced initially, contributing to the early integrity of the healing tendon, although it typically not oriented in parallel [18]. During the third phase, which starts around week six and can continue beyond a year, the extracellular matrix becomes more aligned and collagen I synthesis becomes more prominent [16, 18]. However, the repaired tendon tissue may never fully regain its preinjury properties [19].

The amount of the granulation tissue (callus) can be assessed by the CSA using ultrasound (US) [10, 14] or magnetic resonance imaging (MRI) [20, 21], and the healed tendon is typically considerably larger at the site of the rupture [12]. However, in an animal model, it has also been shown that there is noticeable alteration in the uninjured region far from the rupture site [22], but if such a tissue response occurs in human ATR remains unknown. Previous research has shown that there is fatty infiltration in the calf muscles following ATR [21, 23], and fat infiltration of tendon has also been shown in an animal model [19], but if fatty infiltration of the tendon occurs in humans remains largely undetermined. Reduced loading during Achilles tendon healing yields less matrix formation and altered collagen organization in an animal model [19], but if different rehabilitation protocols, such as delaying the initial loading, affect human tendon morphology remains unknown. Formation of new vessels commonly occurs during the healing process [24], and this may contribute to the overall CSA. This study aimed to explore if the loading regime in the initial 12 weeks after surgical repair of ATR would influence tendon CSA and fat infiltration in the tendon by employing 3D MRI- and US-based metrics. We hypothesized that beginning weight-bearing after 12 weeks (Delayed) would lead to less matrix formation compared with full weight-bearing after 6 weeks (Standard). Furthermore, we hypothesized that the uninjured proximal part of the ruptured tendon would increase in size and that fatty infiltration would occur over time in the Achilles tendon after a rupture.

## 2. Methods

### 2.1. Participants

This study presents secondary analyses of a previously published, prospective, randomized, controlled trial (RCT) comparing how delayed and standard loading following surgical repair of ATR affects tendon healing [21]. The regional Ethics Committee approved the original trial protocol (no. H-19034916). All patients received oral and written information about the trial and patient consent was obtained. The RCT used the following inclusion criteria: diagnosed and surgically treated for an acute ATR within 14 days from injury, no contraindications for MRI, and manage transport to and from the hospital on their own. Exclusion criteria in the RCT were prior ATR in either limb, rerupture, other injuries affecting their lower limb function (such as late sequelae from a complex tibia or fibula fracture, and stroke), anticoagulation treatment, inability to follow rehabilitation or complete follow-ups, smoking, systemic diseases influencing healing, and immunosuppressive treatment including systemic corticosteroid treatment.

**Figure 1:**
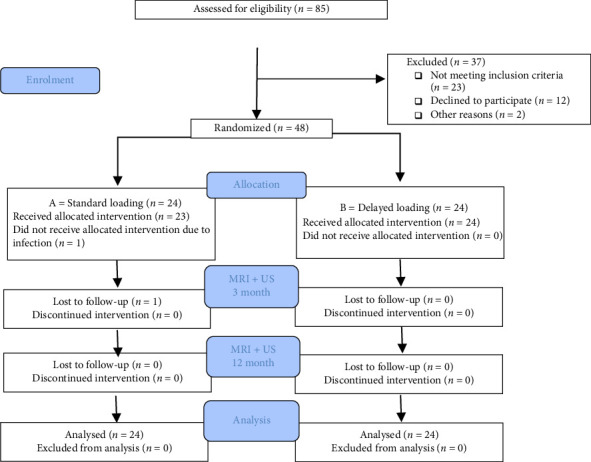
The CONSORT (consolidated standards of reporting trials) flowchart.

MRI measurements were performed on a total of 48 patients by an experienced radiographer (F.H.L.), and US measurements of tendon morphology were performed for the same number of patients by a single experienced physiotherapist (R.H.). At the 3-month follow-up visit, one patient in the Standard group had a deep infection, was treated with silver ions, and was subsequently excluded from the MRI and US tests at this time point (Figure 1: CONSORT flowchart). Patient characteristics are presented in Table 1.

### 2.2. Surgery and Rehabilitation

All patients underwent surgery using a predefined standardized surgical technique involving the Kessler technique for the tendon with a FiberWire 2–0 and closure of the peritenon with Vicryl no. 1 as described previously [21]. The patients were assigned to one of two rehabilitation regimens: the Standard group received the currently accepted rehabilitation including plaster cast for 2 weeks followed by orthosis for 4 weeks and allowed full weight-bearing after week 6. The Delayed group received the same rehabilitation except that the orthosis was used for 10 weeks, initial loading was delayed by 6 weeks, and permitted full weight-bearing after week 12 [21].

### 2.3. MRI

**Figure 2:**
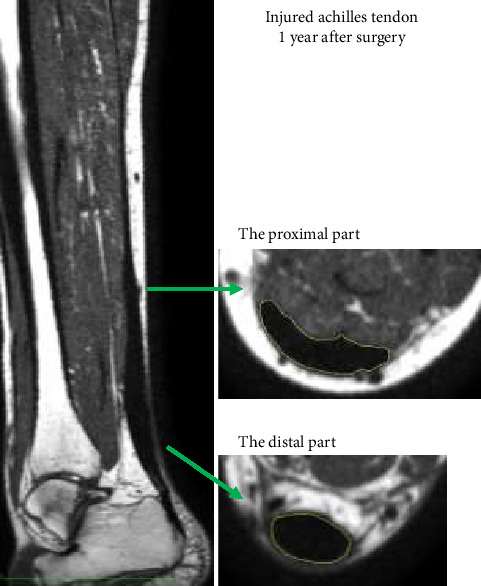
The two different CSA measurement locations. The distal part at the site of the rupture. The proximal at the uninjured part of the tendon.

MRI scans (Philips Ingenia Ambition 1.5T scanner, software Version 5.6.1.2, Netherlands) were performed at weeks 12 and 52. All were scanned in a standard supine position with their legs close together and their feet placed against a plastic foot plate as described previously [21]. A coronal isotropic 3D T1-weighted sequence (voxel size: 0.7 × 0.7 × 0.7 mm^3^) of both ankle and calves was used to measure the CSA of the distal (the free tendon) and proximal parts of the Achilles tendon. An axial 6-point DIXON sequence was applied to assess fat fraction of the tendons. Horos v3.3.6. open-source medical image viewer was used to measure CSA at the two different locations. The CSA of the Achilles tendon was measured manually at half of the distance from the most proximal insertion on the calcaneus and to the most distal part of the myotendinous junction (MTJ) of the soleus muscle (distal part) and of the medial gastrocnemius muscle (proximal part) (Figure 2). Both the injured and uninjured sides were analyzed. DIXON data were upscaled and reoriented to match the 3D sequence using Fiji/ImageJ2 (Version 2.3.0), and the regions of interest (ROIs) from the CSA measurements were used for extracting DIXON fat fraction in each region. Due to a blooming effect of the high subcutaneous fat signal, the outer two voxels (1.4 mm) around the ROI were excluded. Due to the small size of the tendon on the uninjured side and its narrow profile in the proximal part, this resulted in insufficient voxels for analysis and consequently fat fraction could only be determined on the injured distal part of the tendon.

### 2.4. US

All patients had US examinations including Power Doppler flow measurements of the Achilles tendon at weeks 12 and 52. The patient was lying prone with the knees extended and their feet and ankles lying free of the bed. In the area with the highest visible Power Doppler activity in the sagittal plane, two 5-s sine loops (20 frames) were recorded. Fiji/ImageJ2 (Version 2.3.0) was used for quantitative analysis as described previously [21]. These data have been reported previously [21].

### 2.5. Statistical Analysis

GraphPad Prism Version 10.2.2 was used for all analyses. A two-way mixed model (group × time) was used to analyze the tendon CSA and Doppler parameters on the injured tendon. For the fat fraction, only the injured distal part could be analyzed, and data were log transformed due to a log-normal distribution of the raw values. For all tests, *p* ≤ 0.05 was set as the significance level. The difference in CSA between the injured and uninjured tendon was analyzed by a two-way mixed model (side × time).

## 3. Results

Descriptive statistics of Achilles tendon CSA, fat fraction, and power Doppler area (vascularization) at the two time points for the separate groups are presented in Table 2.

### 3.1. MRI

The injured tendon was significantly larger than the uninjured tendon at both time points for both the distal and proximal parts (*p* < 0.0001 for all).

**Figure 3:**
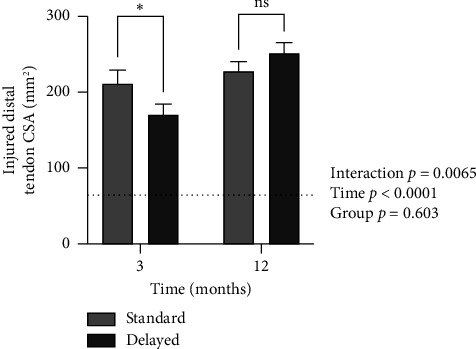
Injured distal tendon CSA in mm^2^ in the Standard and Delayed groups at 3 and 12 months. The mean value for the uninjured side is indicated by the dashed line. Mean ± SEM. ^∗^A significant difference (*p*=0.038) between groups at 3 months in post hoc tests.

**Figure 4:**
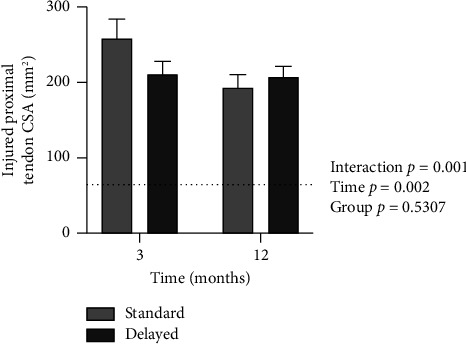
Injured proximal tendon CSA in mm^2^ in the Standard and Delayed groups at 3 and 12 months. The mean value for the uninjured side is indicated by the dashed line. Mean ± SEM.

For the CSA of the distal part, there was a significant interaction (*p*=0.0065) and time effect (*p* < 0.0001) but no group effect (*p*=0.603). In post hoc tests, the distal part was smaller in the Delayed group at 3 months (*p*=0.038), but there was no difference at 12 months. For the CSA of the proximal part, there was a significant interaction (*p*=0.001) and time effect (*p*=0.002) but no group effect (*p*=0.531). Post hoc tests showed no significant group difference at 3 or 12 months in terms of the CSA of the proximal part of the tendon (Figures 3 and 4). The CSAs of the distal and proximal part were measured at a mean distance of 34.7 mm (SD 9.7) and 101.6 mm (SD 11.0) from the calcaneus insertion, respectively.

**Figure 5:**
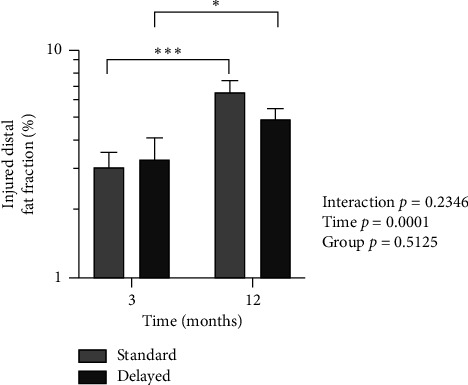
Injured distal tendon fat fraction percentages in the Standard and Delayed groups at 3 and 12 months. Geometric mean and lower-upper SEM. ^∗∗∗^A significant increase in the Standard group from 3 to 12 months (*p*=0.0005) in post hoc tests. ^∗^A significant increase in the Delayed group from 3 to 12 months (*p*=0.045) in post hoc tests.

For fat fraction, there was no interaction or group effect, but there was an effect of time (*p*=0.0001) with a significant increase in fat fraction. Post hoc testing displayed a significant increase in both the Standard and Delayed groups from 3 to 12 months (*p*=0.0005 and *p*=0.045) with no difference between groups (Figure 5).

### 3.2. US

For the Power Doppler activity of the distal part of the tendon, there was a significant interaction (*p*=0.0012), time (*p* < 0.0001), and group effect (*p*=0.006). The Doppler activity decreased in both groups from 3 to 12 months, and at 3 months, the Delayed group had significantly less Doppler compared with the Standard group (Table 2).

## 4. Discussion

The main findings of this study were that at 3 and 12 months, a substantially larger CSA (> 300%) was detected not only at the rupture site but also considerably more proximal to the rupture. In fact, the two portions of the tendon increased with a similar magnitude. The CSA of the injured distal part close to the rupture was 377% while and the proximal part away from the injury site was 308% relative to the uninjured tendon at 12 months. Furthermore, the delayed loading regime yielded less Doppler activity (vascularization) and a smaller CSA at 3 months compared with standard treatment. The 12-month data suggest that there is no disadvantage in delaying rehabilitation initially after surgery, which corroborates our previous results that function, muscle CSA, and tendon length were similar in the two rehabilitation groups [21].

Similar alteration in the injured tendon at 12 months has been reported by Saarensilta et al. [12] who measured the CSA with US and found a ratio between injured and uninjured tendon of 389%. However, in contrast to the present study, Saarensilta et al. [12] only obtained CSA measurements in the area of the rupture site. The fact that the CSA of the tendon proximal to the rupture site also increased markedly indicates that the inflammatory healing response extends to the entire tendon and corroborates animal data [22]. Eliasson et al. [20] used MRI to determine the CSA at 12 months and found absolute values around 200–250 mm^2^, which is comparable to the data of the present study.

It is well known that immobilization and age-related inactivity is associated with infiltration of intramuscular fat [25, 26]. It has also been observed that in muscle strain injuries, there is infiltration of fat located at the intersection between muscle and connective tissue [27]. In an animal model, it has been shown that adipose tissue appears in the healing ATR and that the accumulations are more pronounced when the tendon is unloaded [19]. Fat infiltration in human tendon tissue can occur in aging tendons [28]; however, fat infiltration in human tendon following ATR has not been investigated before. The data herein indicate that there is some fat in the tendon 3 months after an ATR. Moreover, there was a significant rise in fat fraction of the distal tendon portion from 3 to 12 months. Unfortunately, the fat fraction analysis could only be carried out on the tendon after excluding the subcutaneous voxels affected by blooming, which was not possible on the uninjured side due to an inadequate number of voxels. This is a limitation as it would have been informative to compare the results with the fat percentage of the uninjured side.

To what extent different rehabilitation protocols affect tendon morphology remains unclear. Here, we compare two protocols: the Standard protocol, which was allowed full weight bearing 6 weeks after the ATR repair, and the Delayed protocol, which was allowed full weight-bearing 12 weeks after the ATR repair. In the early phase (3 months), the Delayed group had a smaller gain in callus (Figure 2) and less Doppler activity (Table 2) compared with the Standard group. Early loading in an animal model has been shown to prolong the inflammatory response, which may impact tendon remodeling [29]. It is possible that the early loading (Standard) amplifies and prolongs the inflammatory response, which is seen here in the CSA and Doppler data. On the other hand, several animal data indicate that the tendon becomes stronger and contain less disorganized collagen with early loading [19]. However, at 12 months, there were no differences in the size of the tendon or Doppler activity between the groups. In fact, the power Doppler signal was very low in both groups, indirectly suggesting that healing of the tendon approaches completion around 1 year. These findings are consistent with a previously published study on metabolic activity and vascularization after surgical repair of an ATR [30].

## 5. Conclusion

At 3 months, the Delayed regime showed less vascularization and lower CSA for the distal part of the tendon compared with the Standard rehabilitation regime; however, these differences had leveled out after 12 months. In addition, there was an accumulation of fat in the distal part of the injured tendon from 3 to 12 months in both groups. Not only the site of rupture but also the entire tendon on the injured side was affected by the inflammatory repair response with an increase in fat infiltration, vascularization, and CSA. Further investigation is required to fully comprehend these findings, as the consequences for physical function are still elusive.

## Figures and Tables

**Table 1:** Characteristics of patients included in the study (*n* = 48).

Characteristics	Standard *n* = 24	Delayed *n* = 24
Male sex, *n* (%)	17 (71%)	18 (75%)
Age, years	36 ± 10	37 ± 9
Height, cm	179 ± 8	179 ± 11
BMI, kg/m^2^	25 ± 3	26 ± 3
Injured side right/left *n*	10/14	12/12

*Note:* Data are presented as the mean ± SD.

Abbreviation: BMI, body mass index.

**Table 2:** Achilles tendon morphology of patients (*n* = 48) at 3 months and 12 months after surgery.

	All (uninjured)	Standard (injured)		Delayed (injured)	
		3 months	12 months	3 months	12 months
CSA, mm^2^					
Distal	64 ± 2	211 ± 18	229 ± 11	171 ± 13	253 ± 12
Prox.	65 ± 1	259 ± 26	194 ± 17	212 ± 16	207 ± 15
Fat fraction (%)					
Distal^a^	—	3.1 [2.6–3.6]	6.5 ± [5.8–7.3]	3.3 ± [2.7–4.0]	5.0 ± [4.6–5.4]
Doppler^b^ area (mm^2^)					
Distal	—	116 ± 11	12 ± 3	64 ± 12	15 ± 4

*Note:* CSA: cross-sectional area and fat fraction measured by MRI of Distal = distal part of the tendon and Prox. = proximal part of the tendon. Uninjured = mean of all uninjured sides at all time points. Injured = mean of injured sides in each group, STANDARD and DELAYED, at 3 months and 12 months.

^a^Geometric mean and [lower-upper SEM]. Power Doppler area of the distal part of the tendon measured by ultrasound mean ± SEM.

^b^These data have been reported on previously [23].

## Data Availability

The data that support the findings of this study are available from the corresponding author upon reasonable request.
